# Supplementary material for: Plant Defense Responses to a Novel Plant Elicitor Candidate LY5-24-2
Source: Int J Mol Sci. 2022 May 11;23(10):5348. doi: 10.3390/ijms23105348 (PMC9140985; doi:10.3390/ijms23105348)
Supplement: Supplementary file 1 [file ijms-23-05348-s001.zip › ijms-1700968-supplementary.pdf]

Plant Defense Responses to a Novel Plant Elicitor Candidate LY5-24-2

Xin Qi[1,2] †, Kun Li[1,2], Lei Chen[1,2], Yue Zhang[1,2], Nailou Zhang[3], Wei Gao[1,2], Yuedong Li[1], Xingzhong Liu[4]*, Zhijin Fan[1,2]*

[1] State Key Laboratory of Elemento-Organic Chemistry, College of Chemistry, Nankai University, Tianjin 300071, P. R. China

[2] Frontiers Science Center for New Organic Matter, College of Chemistry, Nankai University, Tianjin 300071, P. R. China.

[3] State Key Laboratory of Virology, Wuhan Institute of Virology, Chinese Academy of Sciences, Wuhan, P. R. China.

[4] Department of Microbiology, College of Life Science, Nankai University, Tianjin 300071, P. R. China

Running title: AGB1 regulates the GA pathway by interacting with MYB62

*** Correspondence:**
Corresponding Author
fanzj@nankai.edu.cn
liuxz@nankai.edu.cn

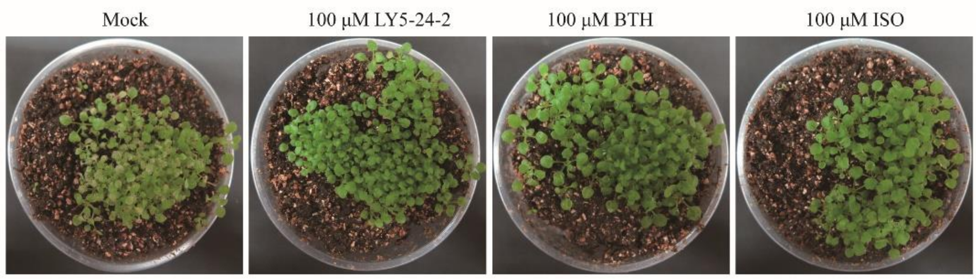

Figure S1. Phenotypic analysis of immune responses induced by LY5-24-2 in *A. thaliana*. Phenotypes of leaves of wild-type (WT) *A. thaliana* inoculated with *Hyaloperonospora arabidopsidis* (*H. arabidopsidis*) Noco2 after sprayed with 100 μM LY5-24-2, BTH, ISO for 24 h. Two-week old *A. thaliana* seedlings were spray-inoculated with *H. arabidopsidis* Noco2. and harvested at 7 d post inoculation.

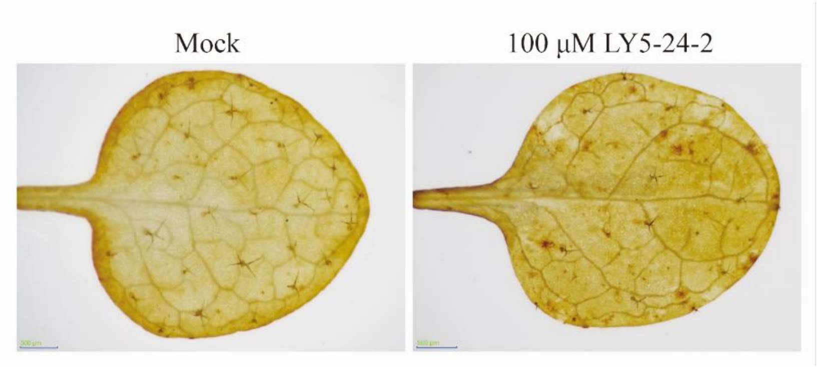

Figure S2. DAB staining in *A. thaliana* at 24 h after a treatment with 100 μM of LY5-24-2. Bars, 2 cm.

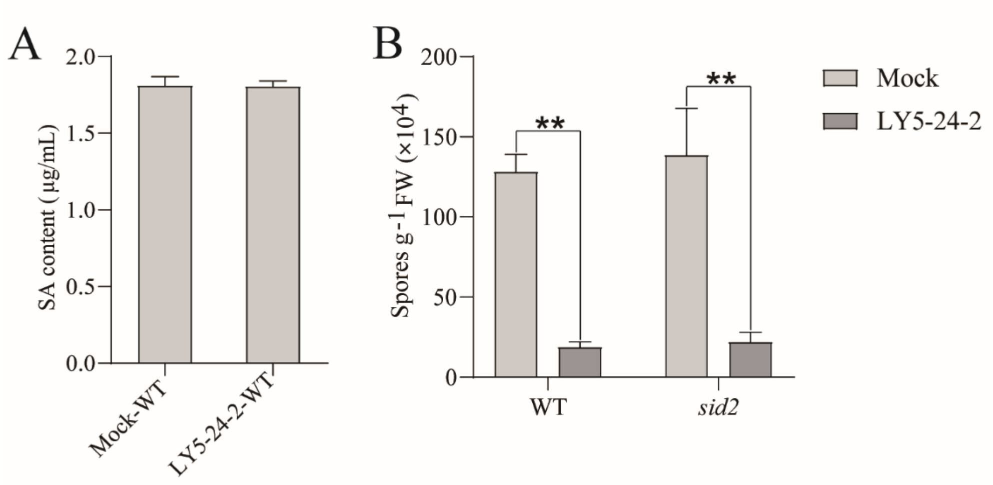

Figure S3. Effect of LY5-24-2 on SA synthesis in *A. thaliana*. (A) SA content analysis at 24 h after a treatment with 100 μM of LY5-24-2. (B) Sporulation level of *H. arabidopsidis* Noco2 on of WT, *sid2* spraying-inoculated with *H. arabidopsidis* Noco2 at 24 h after a treatment with 100 μM of LY5-24-2. Data were shown as the mean of three biological replicates SD (n = 3). The * represent significant difference (*P < 0.05, **< 0.01).

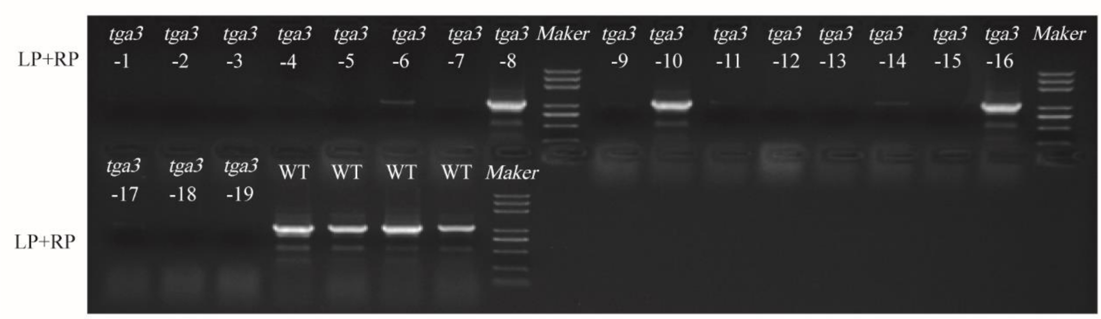

Figure S4. Genomic PCR verification of *tga3* mutant with LP and RP primers.

Nineteen strains of *tga3* (SALK_088114C) mutant of *A. thaliana* were selected, they were obtained by purchasing from the AraShare website (https://www.arashare.cn/index/) and were identified by genomic PCR. The strains

including *tga3*-1 to *tga3*-5, *tga3*-9, *tga3*-11 to *tga3*-15 were selected for subsequent phenotypic analysis based on the genomic PCR results of 5" primer LP and 3" primer RP for *TGA3* gene and the genomic PCR results of 5" LB for T-DNA insertion vector and 3" primer RP for *TGA3* gene[25].

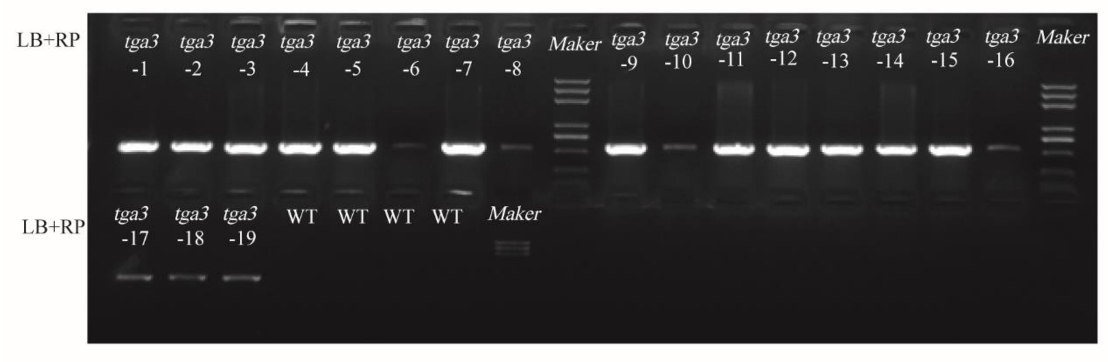

Figure S5. Genomic PCR verification of *tga3* mutant with LB and RP primers.

Nineteen strains of *tga3* (SALK_088114C) mutant of *A. thaliana* were selected, they were obtained by purchasing from the AraShare website (https://www.arashare.cn/index/ accessed on 9 September 2020) and were identified by genomic PCR. The strains including *tga3*-1 to *tga3*-5, *tga3*-9, *tga3*-11 to *tga3*-15 were selected for subsequent phenotypic analysis based on the genomic PCR results of 5" primer LP and 3" primer RP for *TGA3* gene and the genomic PCR results of 5" LB for T-DNA insertion vector and 3" primer RP for *TGA3* gene[25].

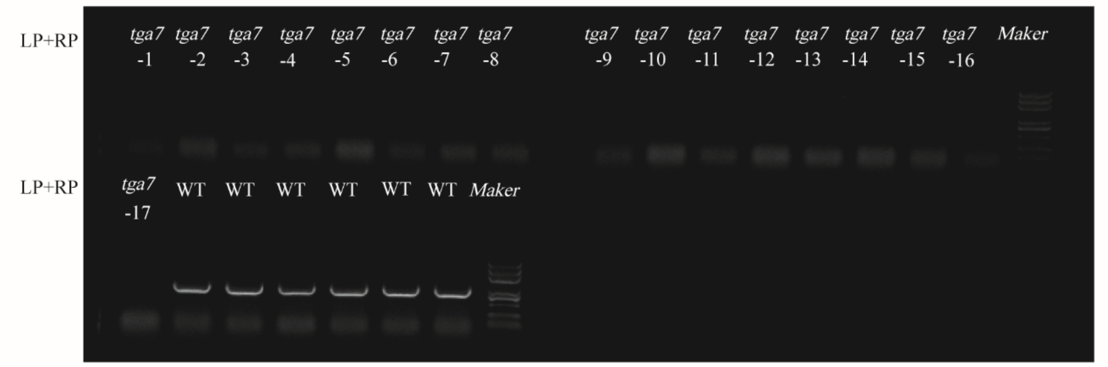

Figure S6. Genomic PCR verification of *tga7* mutant with LP and RP primers.

Nineteen strains of *tga7* (SALK_114488.1) mutant of *A. thaliana* were selected, they were obtained by purchasing from the AraShare website (https://www.arashare.cn/index/) and were identified by genomic PCR. The strains including *tga7*-1 to *tga7*-17 were selected for subsequent phenotypic analysis based on the genomic PCR results of 5" primer LP and 3" primer RP for *TGA7* gene and the genomic PCR results of 5" LB for T-DNA insertion vector and 3" primer RP for *TGA7* gene[25].

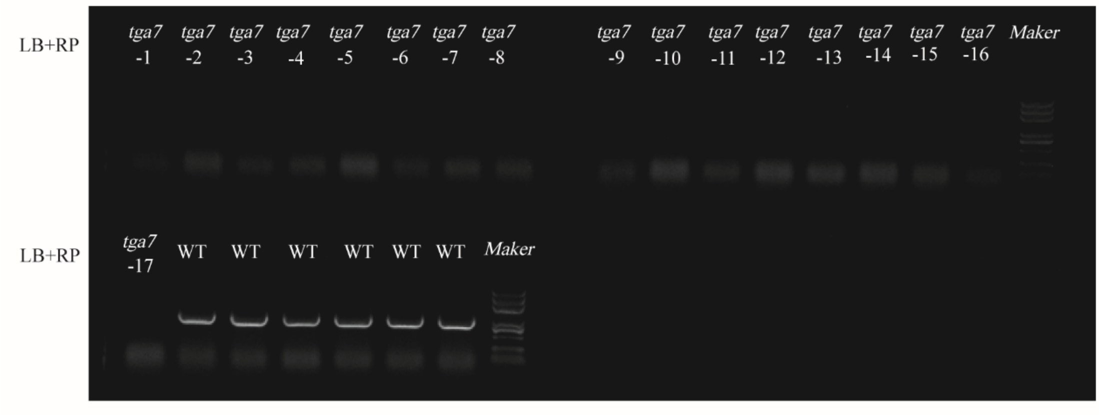

Figure S7. Genomic PCR verification of *tga7* mutant with LB and RP primers.

Nineteen strains of *tga7* (SALK_114488.1) mutant of *A. thaliana* were selected, they were obtained by purchasing from the AraShare website (https://www.arashare.cn/index/) and were identified by genomic PCR. The strains including *tga7*-1 to *tga7*-17 were selected for subsequent phenotypic analysis based on the genomic PCR results of 5" primer LP and 3" primer RP for *TGA7* gene and the genomic PCR results of 5" LB for T-DNA insertion vector and 3" primer RP for *TGA7* gene[25].

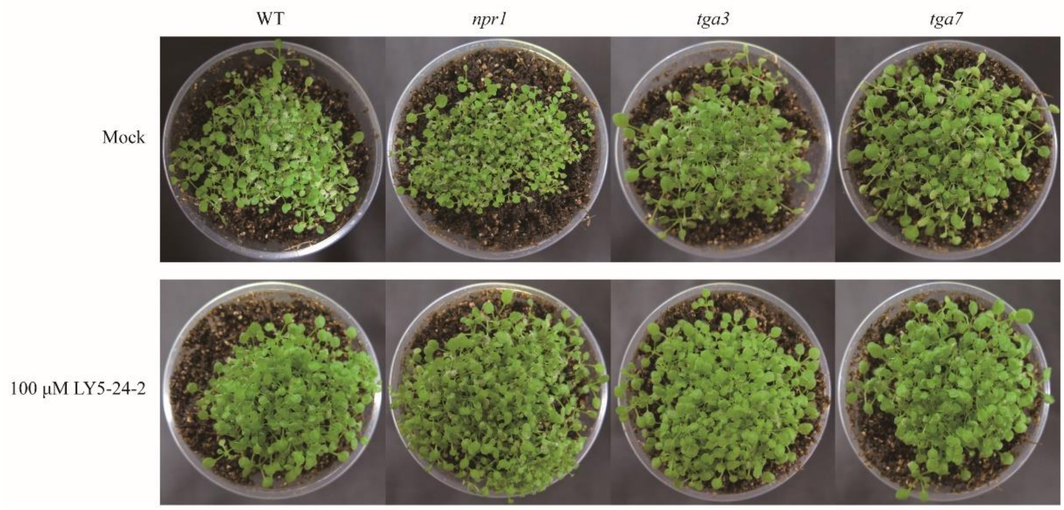

Figure S8. Phenotypic analysis of immune responses induced by LY5-24-2 in *npr1* mutant, *tga3* mutant and *tga7* mutant. Phenotypes of leaves of wild-type (WT), *npr1*, *tga3* and *tga7* inoculated with *H. arabidopsidis* Noco2 after sprayed with 100 μM LY5-24-2 for 24 h. Two-week old *A. thaliana* seedlings were spray-inoculated with *H. arabidopsidis* Noco2. and harvested at 7 d post inoculation.

Table S1. Primer sequences used in this study.

Primer name	Primer Sequence
AtTUB4-F	CGAAAACGCTGACGAGTGTA
AtTUB4-R	CCTTGGGAATGGGATAAGGT
RT-AtNPR1-F	TTGTTTATCTGGCCGCCGAA
RT-AtNPR1-R	ACAAGCTTAGCGTCGCTGTA
RT-AtPR1-F	TCCCTCGAAAGCTCAAGATA
RT-AtPR1-R	GTTGCCTCTTAGTTGTTCTG
RT-AtPR2-F	TAGCTTCCTTCTTCAACCAC
RT-AtPR2-R	CCATCTCTGTAGCTCTGAAC
RT-AtPR3-F	GTTTCTACACTTACAACGCC
RT-AtPR3-R	ATCCACCTGTAGTTTCATGG
RT-CsNPR1-F	CGCTTTGGTGAGGAGTTT
RT-CsNPR1-R	CAAGATTACAACAAGGGT
RT-CsPR4-F	TGGGATGCTAACAAGCCT
RT-CsPR4-R	CCGTTGGAGCATTGATCA
RT-CsPR5-F	CTTCTGCTAGTTGTGTTG
RT-CsPR5-R	GCAGTCACCCGTCTGGCA
RT-CsCAT-F	ATGCTGGAAGAGGAGGCTAT
RT-CsCAT-R	ATGGTGAGGACATTTGGGAG
RT-CsAPX-F	ATGGGAAAGTGCTACCCTGTT
RT-CsAPX-R	ACAATGTCCTGGTCCGAAAG
RT-CsActin-F	GTTACGCCCTCCCTCATGCCATTC
RT- CsActin-R	TCCCGTTCGGCAGTGGTGGT
tga3-LP	CTGCATAGCACTGAGACCCTC
tga3-RP	GAAAACCCAGCTCTCCAAAAC
tga7-LP	ACATCCTCAAACGTGAGCATC
tga7-RP	TTGGATGGATATGCTACCGAG
LB1.3	ATTTTGCCGATTTCGGAAC

Table S2. In vitro fungicidal activity of LY5-24-2 a concentration of 50 mg/L (%).

Fungi	LY5-24-2	ISO
A.s (*Alternaria solani*)	34.4%	4.8%
B.c (*Botrytis cinereal*)	46.8%	51.5%
C.a (*Cercospora rachidicola*)	28.0%	36.8%
G.z (*Gibberella zeae*）	43.4%	4.4%
P.p (*Physalospora piricola*)	36.0%	31.8%
P.s (*Pelliculariasasakii*)	44.0%	52.1%
P.z (*Phytophthora infestans* (Mont.) de Bary)	21.4%	17.9%
R.c (*Rhizoctonia cerealis*)	75.5%	53.5%
S.s (*Sclerotinia sclerotiorum*）	79.5%	63.6%

Reference

25 Qi, X.; Chen, L.; Zhang, Y.; Gao, W.; Chen, L.; Wang, D.; Tang, L.; Wang, Z.; Wang, N.; Fan, Z. The methoxyacrylate fungicide candidate CL-15C also functions as a plant elicitor in *Arabidopsis thaliana* and *Oryza sativa* L. *J. Agric. Food Chem.* **2022,** *70*, 3142–3150. https://doi.org/10.1021/acs.jafc.1c07757.
